# On Some Aspects of Memory

**Published:** 1901-03-23

**Authors:** John Aikman


### On Some Aspects of Memory.

By John Aikman, M.D.

III.—ARCHITECTURE—(*concluded*).

In the same category as "worry" comes "doubt." The reverence of Agnosticism does not exist in doubt. The Agnostic rests upon memory confirmed by judgment and waits "As he stands on the heights of his life, with a glimpse of a height that is higher." (Tennyson, "By an Evolutionist.")

The doubting mind has no resting place. It gropes blindly among what is uncertain, until it must cry, "It is all barren; and so it is, and so is all the world to him who will not cultivate the fruits it offers." (Sterne, "Sentimental Journey.")

And these frames of mind are habits. They are under control until they become so. Education and experience give the calmness which characterises the academic mind. "Man the sufferer, by careful service, becomes the interpreter of Nature. Man, the interpreter, becomes the Master." And his progress towards mastery is written not only in his brain cells. His features change their form from the emotions which disturb them. Both men and animals can read that record.

"Husband and wife come to look alike at last, as has often been noticed. It is a common saying of a jockey that he is 'all horse,' and I have often fancied that milkmen get a stiff upright carriage, and an angular movement of the arm that remind one of a pump, and the working of its handle" (Wendell Holmes, "Professor at the Breakfast Table," p. 120).

In such playful terms Oliver Wendell Holmes describes this feature record.

The author of the "Choir Invisible" touches a fuller chord: "Years upon years of true thoughts, like ceaseless music shut up within, will vibrate along the nerves of expression until the lines of the living instrument are drawn into correspondence, and the harmony of visible form matches the unheard harmonies of the mind" (p. 346).

The feature record is not what we might have done, but what we have done. It is slowly formed; it is yearly changing. It evidences, not the power of the instrument, but the part it has played. In those who are well known to us it provokes joy or pity. In new acquaintances it provokes criticism, based upon the association of former memories. We have to caution ourselves not to be guided by first impressions. Yet first impressions run along the most established memory paths. They are established, but they are not final. They are worthy of reverence only while they are under the control of a fuller judgment.

Even the experienced are misled. Take an instance from the workroom of a London consulting physician. A broken-down man came for advice as to his health. The physician, after careful examination, told him that his health was good, and that he only required a lighter view of life to make him happy. "Go and see Grimaldi," he said, "He will cure you, he will make you laugh." The reply was, "Sir, I am Grimaldi."

Memory makes the instrument. In the past it is influential history. In the present it is association and motive. Into the future it casts suggestions and imagination. For round the Real hangs the Ideal.

Before I trace that relationship, I wish to make clear that while we have free will to judge we have fond will to pervert judgment. The reason is not far to seek. Some large number of our brain cells have no higher destiny than to maintain ourselves in life. These cells receive the earliest and the deepest imprint. They are fed, during disease, when our memory cells are left to starve. They are the strong and high-handed members of the community. We could not live if it were otherwise. Greed is the factor of our animal life. Such an environment is dangerous to cells which are capable of a higher development. Health and our prerogative of free will save us from what is ignoble.

Memory makes the instrument, and we make memory by a reverent attention. Let me quote a recent description of a good instrument. "His keenness and significance were the outcome, not of any cool eye to the main chance, but of a gay sense of the pure need of logic, not only in letters, but also in living." ("Capetown to Ladysmith," G. W. Steevens. Mr. Vivian Blackburn, note, p. 149.)

It is that pure need of logic in living which distinguishes success from failure. Logic is a development of the justness of memory. The mind at its best is characterised by the truth, balance, and justness of memory. Our best physiognomy is the record of how much these are happy memories. Our faulty record shows how far we have stifled them with a selfish eye to the main chance.

For example, justice follows upon fact as a natural consequent. It is strong, it is universal. Revenge, the wild justice of Bacon, follows a memory which is personal, limited in conception, and with a heavy lurch towards error. In both instances the chord touched is real. The emotion excited depends upon the instrument. And when we examine the instrument we find that it is

> The sin that practice burns into the blood
> And not the one dark hour which brings remorse,
> Will brand us after, of whose fold we be.
> —(Tennyson, "Merlin and Vivien.")

Justice leans upon memory. Revenge rushes from it.

The same principle rules our success in all walks of life. From the memory which we have made for ourselves we forecast our suggestions and imaginations, with a speculative hope that we shall increase our store. In the exercise, as in the building of our faculties—

> Memory is the warder of the brain.
> —(*Macbeth*, Act I., Scene 2.)

As the inventor leans upon memory he succeeds, perhaps, after many disappointments. When he leaves the foothold of memory his way becomes insecure.

The poet gives us—

> Wisdom wedded to immortal verse.
> —(Wordsworth, "Excursion," Bk. vii.)

Like the novelist, he must link the memory of the past with association and motive in the present.

The painter draws his inspiration from memory. His imagination is anchored to, rather than fettered by, its associations. His nerve sheaths are strong and full of life. They prepare him to see things which other men miss, and he becomes a prophet—in the Carlylean sense —"a seer." He teaches us what to seek, and we find it. No.

> Apt alliteration's artful aid
> (Churchill, "Prophecy of Famine")

covers his faults. But in fixed line there is movement, and in fast colour there is change; the record of nature's permanence and variation.

I instance these classes of men and women, inventors, poets, novelists, and painters, because the evolution of memory becomes supreme in their lives under the influence of a patient judgment.

What can I say of failure?

Dr. Savage, in a recent lecture upon "Mental Dissolution" (*Lancet*, February 10th, 1900, page 361), tells us that "the memory of the long past may appear with unusual vividness. So that there is, or appears to be, an increase in exactness in the memory of the long past." The recent blur, and its misinterpretation, leads to the evolution of foolishness. This means that during early life, and in the graver moments of more advanced life, millions of brain cells have undergone evolution towards that which is precise and true. These pathways at least are brain capital of sterling worth; and

It becomes no man to nurse despair,
But, in the teeth of clenched antagonisms,
To follow up the worthiest till he die.
—(Tennyson, "The Princess.")

And this sketchy, imperfect history of our brain mechanism teaches no new lesson. Truth, carefulness, thoroughness, and earnestness have always characterised the best of men. Dante, in his memorable journey with Virgil, learnt of the waters of Purgatory. Lethe, the water of forgetfulness.

But lo, where Eunoe flows,
Lead thither: and, as thou art wont, revive
His fainting virtue.
—(Dante, "Purgatory," Canto XXXIV.)

And Thomas Moore, nearer our own time, gives us the song of the two cupbearers. The first sings:—

Drink of this cup—Osiris sips
The same in his halls below;
And the same he gives, to cool the lips
Of the dead, who downwards go.
Drink of this cup—the water within
Is fresh from Lethe's stream;
"'Twill make the past with all its sin,
And all its pain and sorrow seem
Like a long forgotten dream!"

And the second cupbearer sings:—

Drink of this cup—when Isis led
Her boy of old to the beaming sky,
She mingled a draught divine, and said,
"Drink of this cup, thou'lt never die!"
And memory, too, with her dreams shall come
Dreams of a former, happier day,
When heaven was still the spirit's home
And her wings had not yet fallen away.
Glimpses of glory, ne'er forgot,
That tell, like gleams on a sunset sea,
What once hath been, what now is not,
But, oh! what again shall brightly be!"

Choose which you will.

